# Transmission of Influenza on International Flights, May 2009

**DOI:** 10.3201/eid1707.101135

**Published:** 2011-07

**Authors:** A. Ruth Foxwell, Leslee Roberts, Kamalini Lokuge, Paul M. Kelly

**Affiliations:** Author affiliations: Department of Health and Ageing, Canberra, Australian Capital Territory, Australia (A.R. Foxwell, L. Roberts);; Australian National University, Canberra (A.R. Foxwell, L. Roberts, K. Lokuge, P.M. Kelly);; ACT Government Health Directorate, Canberra (P.M. Kelly)

## Abstract

Understanding the dynamics of influenza transmission on international flights is necessary for prioritizing public health response to pandemic incursions. A retrospective cohort study to ascertain in-flight transmission of pandemic (H1N1) 2009 and influenza-like illness (ILI) was undertaken for 2 long-haul flights entering Australia during May 2009. Combined results, including survey responses from 319 (43%) of 738 passengers, showed that 13 (2%) had an ILI in flight and an ILI developed in 32 (5%) passengers during the first week post arrival. Passengers were at 3.6% increased risk of contracting pandemic (H1N1) 2009 if they sat in the same row as or within 2 rows of persons who were symptomatic preflight. A closer exposed zone (2 seats in front, 2 seats behind, and 2 seats either side) increased the risk for postflight disease to 7.7%. Efficiency of contact tracing without compromising the effectiveness of the public health intervention might be improved by limiting the exposed zone.

The emergence of pandemic influenza A (H1N1) 2009 in Mexico and the United States, with rapid spread to Europe, Asia, and the Pacific, is testament to the ease of spread of infectious disease across the globe (*1*). The World Health Organization activated level 5 pandemic alert on April 29, 2009, when sustained community transmission of the pandemic virus was demonstrated in Mexico and the United States. In her address to the United Nations on May 4, 2009, Margaret Chan, Director-General of the World Health Organization, called for heightened vigilance to limit international spread of the virus (*2*). Australia’s response was rapid, with the introduction of a number of measures as outlined in the Australian Health Management Plan for Pandemic Influenza, 2008 (*3*). These measures included in-flight messages to incoming passengers, use of health declaration cards by all incoming travelers, and mandatory reporting by the pilot on the health status of crew and passengers before landing (*4*). The novel virus was also listed as a quarantinable disease under Australia’s Quarantine Act 1908, which allows for the application of public health powers for intervention (*5*).

Reports documenting spread of disease during airline flight are limited (*6**–**9*). Specific policy stating that passengers sitting in the same row as and within 2 rows of a confirmed case-patient should be treated as suspected of having that disease relies on studies of air travel where the index case-patient was infected with *Mycobacterium tuberculosis* (*10**–**12*). The aim of this study was to investigate the spread of pandemic (H1N1) 2009 infection from persons with confirmed disease on flights to Australia during May 2009. The spread of other influenza-like illness (ILI) was also documented.

## Methods

### Study Population

A retrospective cohort study designed to determine exposure risk to known pandemic (H1N1) 2009 virus was undertaken for 2 long-haul flights that entered Australia the weekend of May 23–24, 2009. Flight 1 was chosen after identification of 6 passengers with confirmed pandemic (H1N1) 2009 infection within 24 hours after flight arrival from the United States. Flight 2 was chosen after identification of a confirmed case of pandemic (H1N1) 2009. This flight came from an area that lacked community transmission. Passenger details were obtained through collection of Health Declaration Cards and comparing the cards to flight manifests obtained from the airlines.

The definition of ILI was broad to capture as many persons as possible within the dataset. Passengers were asked to self-report development of any of the following signs or symptoms: fever, cough, sore throat, headache, runny nose, muscle aches, diarrhea, and lethargy. ILI was defined as >1 symptom (cough, runny nose, sore throat, or fever) within 7–14 days before the flight or during the flight or <7 days after arrival. The time periods were put in place to help determine when passengers were most likely to have contracted their ILI. Passengers were excluded who indicated bacterial infection (antibiotic prescription from medical personnel) or regular health issues, such as migraines. All but 4 passengers reporting symptoms had confirmation of ILI status from a qualified health professional.

Self-identification of other health conditions that were considered potential concurrent conditions for purpose of this study included obesity, diabetes mellitus, immunosuppression, asthma, chronic lung disease, and pregnancy. Seat location, concurrent condition status, and contraction of disease were compared. Ethics approval was given by the Australian Government Department of Health and Ageing Ethics Committee and the Australian National University Human Research Ethics Committee.

### Data Collection

Surveys were distributed to passengers 3 months after flight arrival. The survey asked about influenza-like symptoms, symptom onset time, concurrent conditions, antiviral prophylaxis and treatment, isolation or quarantine dates, other potential exposure to ILI before and after the flight, contact with health professionals after the flight, and details of testing for pandemic (H1N1) 2009 virus. Two reminders were sent to improve the response rate of the study.

As a triangulation method, all passenger names, passenger sex, disease onset dates, and postal codes were cross-checked against those of passengers with known pandemic (H1N1) 2009 cases that were notified to national authorities for 1 month after flight arrival to verify information received from the survey responses and identify additional cases. Travel details were verified for pandemic (H1N1) 2009 case-patients identified through national notification. Contact tracing through public health authorities also identified ILI case-patients who had negative laboratory test results for pandemic (H1N1) 2009 virus.

### Data Analysis

The increased risk of passengers contracting either laboratory-confirmed pandemic (H1N1) 2009 or an ILI (including pandemic [H1N1] 2009) was separately estimated by dividing the number of persons with the illness by the number of susceptible persons in the contact zones as described. For pandemic (H1N1) 2009, only passengers sitting in the economy class were considered exposed because of the location of persons displaying symptoms preflight or during the flight and the sectional layout of the aircraft.

## Results

Of the 738 passengers on the 2 flights, 319 (43%) responded to a questionnaire; 143 (18%) passengers could not be contacted. Cross-checking of mandatory notifications of pandemic (H1N1) 2009 against all passengers on both flights found 2 additional pandemic (H1N1) 2009 infection cases while also confirming symptom data on survey responses. Contact tracing by public health authorities found 5 additional passengers with ILI who had negative test results for pandemic (H1N1) 2009.

No passengers who had positive test results for pandemic (H1N1) 2009 had underlying health conditions considered to make them more susceptible to influenza; however, 5 of 32 passengers reporting ILI postflight had >1 potential concurrent conditions (1 each of obesity, diabetes mellitus, pregnancy, asthma, and chronic lung disease). Limited analysis demonstrated that the concurrent condition did not make these persons more susceptible than other passengers to contracting an ILI.

### Flight 1

Flight 1, an Airbus A380, embarked from Los Angeles and arrived in Sydney on May 24, 2009, carrying 445 passengers. Of the 188 (42%) passengers who responded to a survey, 169 (90%) were Australian residents. Response rate varied with class of travel, with 11 (79%) of 14 first class passengers, 40 (56%) of 71 business class passengers, 19 (59%) of 32 premium economy class passengers, and 117 (36%) of 327 economy class passengers responding.

Combined results from the survey and disease notification data sources identified 8 passengers who had an ILI at the beginning of the 14-hour flight. For 4 of these passengers, pandemic (H1N1) 2009 infection was later laboratory confirmed; for 1, pandemic (H1N1) 2009 infection was confirmed as negative; 3 passengers were not tested. ILI symptoms developed in 2 other passengers during the flight, and pandemic (H1N1) 2009 was confirmed in both.

**Figure 1:**
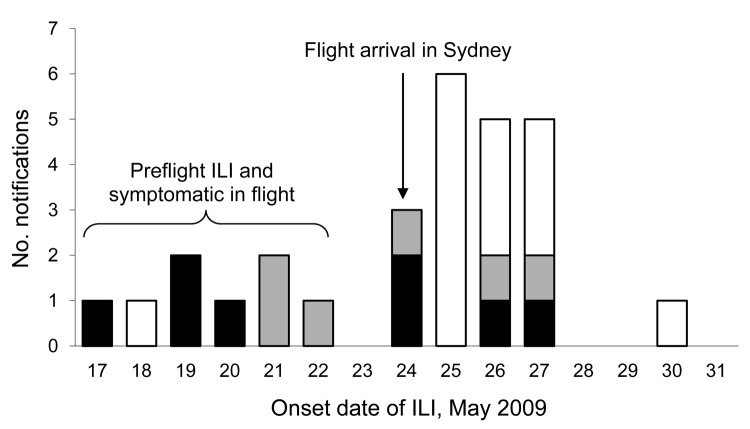
Onset date of influenza-like illness (ILI) in passengers traveling to Australia on flight 1, May 24, 2009. Six other passengers did not state exact ILI onset date. White bar sections indicate a negative test result for pandemic (H1N1) 2009 virus; black bar section indicates a positive test result for pandemic (H1N1) 2009; gray bar sections indicate ILI with no test given.

Twenty-four passengers were identified as developing ILI symptoms <7 days after arrival in Australia. Of these, 2 passengers had laboratory-confirmed pandemic (H1N1) 2009 infection, 15 had illness confirmed as negative for the pandemic virus, and 7 were not tested. Most passengers experienced onset of symptoms <3 days after flight arrival; however, 6 passengers did not state exact date of disease onset (Figure 1).

Self-reporting of symptoms from passengers did not distinguish between different causes of ILI (Table). Fever was reported from 4 of 8 passengers with confirmed pandemic (H1N1) 2009, 7 of 16 passengers whose ILI was confirmed negative for pandemic (H1N1) 2009, and 2 of 10 passengers with ILI who were not tested. Two or fewer symptoms, not including fever, were reported for 3 of the 8 passengers with confirmed pandemic (H1N1) 2009, 7 for those testing negative for pandemic (H1N1) 2009, and 6 with ILI who were not tested.

**Table:** Signs and symptoms reported by passengers on flight 1 with influenza-like illness, by pandemic (H1N1) 2009 testing status and results, Australia, May 2009*

Sign or symptom	Tested		Not tested, n = 10
	Positive, n = 8	Negative, n = 16	
Fever	4	7	2
Cough	6	8	3
Sore throat	1	9	6
Headache	4	8	3
Runny nose	5	10	5
Muscle aches	3	6	3
Diarrhea	1	2	1
Lethargy	5	7	2
Vomiting	0	1	0

*Signs and symptoms are not mutually exclusive.

#### Location and Transmission

**Figure 2:**
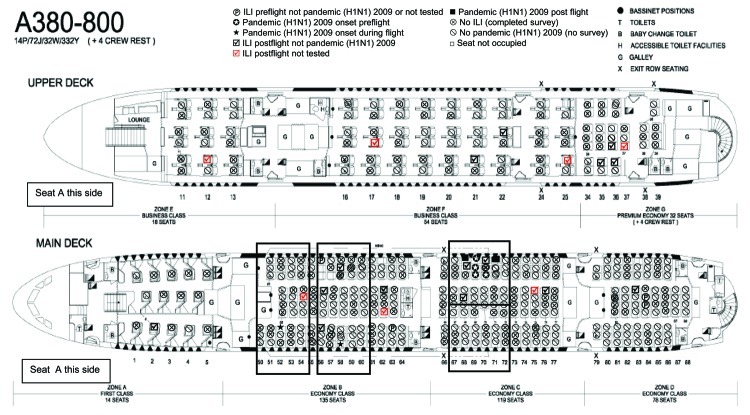
Passenger configuration on flight 1 arriving in Sydney, New South Wales, Australia, on May 24, 2009. ILI, influenza-like illness.

Twenty (83%) of 24 passengers in whom an ILI developed postflight sat in aisle seats (Figure 2). This seat location increased the risk of contracting an ILI by 1.8×; however, it did not reach statistical significance. Survey respondents were 1.3× more likely to sit in an aisle seat.

Some clustering of cases was seen with the potential for spread of either ILI or pandemic (H1N1) 2009 from passengers who were infectious before and during the flight (Figure 2). The passengers who were symptomatic for pandemic (H1N1) 2009 before the flight departed were traveling as a family group; however, they were not connected to the persons who contracted the virus during the flight. Likewise, 2 other clusters of nonpandemic ILI with either symptomatic persons boarding the flight or showing symptoms postflight were in family groups and therefore would have had substantial contact before, during, and after the flight. Disease was deemed to have spread during the flight in passengers from seats 69H/69J/69K/70H to 68K and 71K, with all case-patients having positive test results for pandemic (H1N1) 2009; from 63C to 62D (neither passenger being tested); and from 84E/84F to 83G, with all case-patients testing negative for pandemic (H1N1) 2009. One cluster (seats 35B/36B/36D/37D) had no identifiable preflight index case-patient; however, 2 of the passengers were part of a family group.

In the cabin section of rows 66–77 was a cluster of passengers with symptoms before boarding who were later found to have positive test results for pandemic (H1N1) 2009. ILI symptoms developed in 7 passengers after the flight. In 2 of these passengers, pandemic (H1N1) 2009 was laboratory confirmed; for 4, ILI was confirmed as negative for pandemic (H1N1) 2009; and 1 person (seat 75G) was not tested.

Similarly, in the cabin section of rows 50–64, symptoms developed in 2 passengers during the flight or on the day of arrival (seats 52C and 58B); these passengers were found to be pandemic (H1N1) 2009 positive. Symptoms developed in 7 passengers after flight arrival; 5 of whom had negative test results for pandemic (H1N1) 2009, 2 (seats 54F and 62D) were not tested.

#### Risk of Contracting Disease and Contact Tracing

We examined the risk of contracting pandemic (H1N1) 2009 infection postflight to all susceptible passengers seated in the economy section of the aircraft. Health authorities contacted 145 passengers on flight 1 for quarantine and prophylactic treatment after potential exposure to pandemic (H1N1) 2009 (Figure 2, large black boxes). Of these, 52 (35%) passengers responded to the surveys, of whom 8 (15%) went into isolation 1 day after flight arrival, 17 (33%) by 2 days after flight arrival, and the others >3 days after flight arrival.

Initially, we looked at passengers exposed to this disease who sat in the same row as or within 2 rows either side of passengers who had symptoms develop before or during the flight. The increased risk of contracting pandemic (H1N1) 2009 by sitting in those seats was 1.4% (95% confidence interval [CI] –0.5% to 3.4%). If the contact zone was modified to sitting in the same row as or within 2 rows either side of passengers who had preflight symptoms (rows 67–72) and not those in whom symptoms developed during the flight, the risk of contracting pandemic (H1N1) 2009 postflight increased to 3.6% (95% CI –1.3% to 8.6%). No passengers were detected as acquiring pandemic (H1N1) 2009 from either of the passengers with symptoms that developed during the flight; however, 2 passengers in the same section of the aircraft who responded to the survey indicated having had ILI symptoms but not being tested for pandemic (H1N1) 2009.

The current zone for contact tracing is defined by passengers in the same row as and within 2 rows either side of the index case-patient. A closer zone forming a square delimited by 2 seats in front, 2 seats behind, and 2 seats on either side of the index case-patient could be prescribed (Figure 2, small black boxes [rows 67–72]). When the risk of contracting pandemic (H1N1) 2009 postflight was calculated for economy passengers sitting in the 2 × 2 square around the preflight symptomatic index case-patients in seats 69H–70H, the risk of becoming ill postflight increased to 7.7% (95% CI –2.6% to 17.9%).

### Flight 2

On May 23, 2009, a Boeing 747-400 arrived in Sydney from Singapore carrying 293 passengers. Of the 131 (45%) passengers responding to a survey regarding the potential for contracting an ILI during their flight, 114 (87%) were Australian residents. Public health authorities were not alerted to the potential for passengers carrying pandemic (H1N1) 2009 virus on this flight until 6 days after arrival. They were alerted after mandatory notification of the virus infecting 1 passenger.

Survey data showed that 1 passenger was identified as having symptoms consistent with an ILI before the flight and that symptoms developed in 2 additional passengers on the flight. The passenger who was symptomatic before the flight was not tested for pandemic (H1N1) 2009; of the passengers whose symptoms developed during the flight, 1 was not tested and 1 (adult in seat 33D) was tested 6 days after flight arrival. At that time, the test returned a negative result.

**Figure 3:**
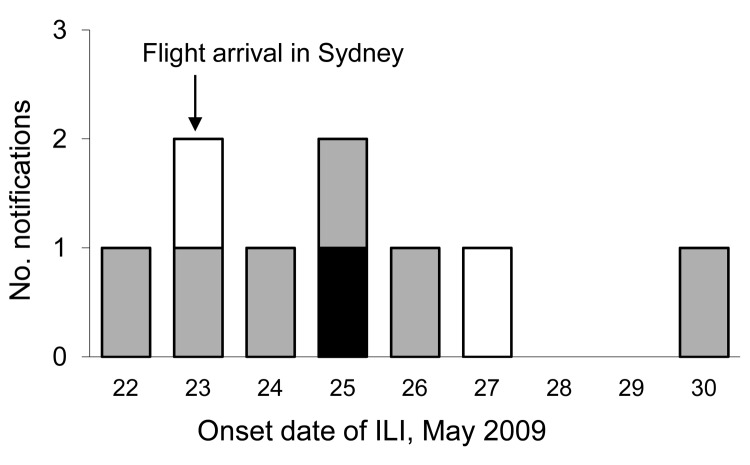
Onset date of influenza-like illness in passengers traveling to Australia on flight 2, May 23, 2009. White bar indicates a negative test result for pandemic (H1N1) 2009 virus; black bar indicates a positive test result for pandemic (H1N1) 2009; gray bars indicate ILI with no test given. ILI, influenza-like illness.

Six passengers were identified from the survey as having ILI symptoms within 7 days after flight arrival (Figure 3). Of these, 1 passenger with symptom onset within 48 hours after flight arrival (a child in seat 33D) was laboratory confirmed to have pandemic (H1N1) 2009, 1 passenger (seat 24K) had negative test results for pandemic influenza A, and 4 passengers (seats 34A, 35B, 41G, and 63D) were not tested. The passenger in whom pandemic (H1N1) 2009 was confirmed was a child companion sharing a seat on the aircraft with the passenger whose symptoms developed during the flight but who tested negative for the virus.

Some clustering of cases was seen, with a potential spread of disease from passengers who were symptomatic during the flight (seats 62C–63D, 33D–34A, and 35B and the child sharing 33D). Transmission of disease between adult and child in 33D could have occurred before or during the flight. With lack of a confirmed index case-patient for pandemic (H1N1) 2009, the increased risk for disease was not calculated. No other passengers on this flight had positive test results for pandemic (H1N1) 2009 infection after triangulation methods with the notifiable diseases database.

## Discussion

Of 2 long-haul flights entering Australia within the first month after declaration of a level 5 alert for pandemic (H1N1) 2009, a total of 45 (6%) of 738 passengers on 2 aircraft were identified as having the potential to spread an ILI into the local community. Follow-up confirmed 9 passengers with pandemic (H1N1) 2009 infection; 8 of these were from flight 1.

Flight 1, originating from a destination with documented widespread community transmission of pandemic (H1N1) 2009, had the greatest potential for introducing the pandemic virus into the Australian community, with 2% of its tested passengers being confirmed positive for pandemic (H1N1) 2009. Spread of the virus from a region known to have sustained community transmission was to be expected and formed part of the case definition in Australia during the early phases of the pandemic (*13*). However, flight 2 originated in Singapore, where the first recorded case of the disease was on May 26, 2009 (*14*), 3 days after the aircraft arrived in Australia, which suggests that a targeted approach to aircraft screening that relies on country-specific information is not completely reliable.

Transmission of ILIs on board these aircraft clustered closely with a passenger who was symptomatic during the flight or may have been in contact with an infectious passenger for >15 minutes during the flight. This finding is similar to transmission of pandemic (H1N1) 2009 noted on a long-haul flight to New Zealand in 2009 (*6*). Recent studies on the transmission of pandemic (H1N1) 2009 in ferrets demonstrated preference for aerosol and droplet transmission of the virus (*15**,**16*). A similar study investigating the disease in a tour group in China indicated droplet transmission from coughing or talking with the index case-patient as being the main mode of transmission (*17*). The cabin in the A380-800 (flight 1) allows for a 10% wider seat in economy class than does the 747-400 (flight 2) (*18*), and modern ventilation systems in aircraft circulate air around bands of seat rows rather than the through length of the aircraft (*19*). However, neither of these measures are enough to prevent droplet transmission from either talking (≈1 meter) or spread of smaller aerosol droplets (*7**,**8*).

Vigilance by health authorities and cooperation by the public assisted in detecting many ILIs that were not associated with pandemic (H1N1) 2009. These ILIs could be caused by different viruses, as seen by Follin et al. (*20*). Follin et al. reported that, although 5% of the 70 passengers examined in their study had pandemic (H1N1) 2009, rhinovirus, coronavirus, influenza B, and parainfluenza were also detected.

Contact tracing and implementation of public health intervention measures after in-flight exposure to disease is time and resource intensive (*21*). Southern Hemisphere estimates of the serial interval for pandemic (H1N1) 2009 varied from 1.5 days to 2.9 days (*22**,**23*), yet practicalities associated with disease diagnosis and contact tracing meant that quarantine dates began 1–5 days after flight arrival, thus minimizing opportunities to halt transmission by social isolation or chemoprophylaxis. Although compliance with the current practice of following up all passengers in the same row as and within 2 rows either side of the index passenger (*11*) was similar to a recent survey from Switzerland of air travelers in Europe (*24*), the increased risk of contracting disease as found in the current study would suggest that further limiting of the zone required for contacting exposed passengers could assist in efficient yet effective public health outcomes. Furthermore, use of risk assessment of different diseases would enable implementation of a public health response that would be proportionate to potential disease severity.

Pandemic (H1N1) 2009 and other ILIs can be spread to a community by passengers who were symptomatic before boarding the aircraft. Four of 9 of passengers in whom pandemic (H1N1) 2009 was diagnosed displayed symptoms preflight. This finding is similar to that in a recent study looking at the travel patterns of patients with pandemic (H1N1) 2009 reported from Singapore, where 25% of patients had symptoms before boarding their flights (*14*). Modeling predicting the global dynamics of disease spread and evidence obtained during the grounding of flights in the United States after September 11, 2001, demonstrated that travel restrictions can delay the intercity spread of influenza (*25*). Further modeling has shown that the intervention by preventing symptomatic passengers from boarding flights, particularly at airports considered major hubs, assisted in delaying influenza spread by up to 2 weeks (*26*). The control measure of exit screening, combined with the potential value of deterring passengers from travel, also efficiently restricts the spread of other respiratory illnesses (*27*).

Potential limitations in this study include lack of knowledge of the health status of passengers who did not return the survey or inform health authorities of ILI symptoms after flight arrival, response bias resulting from contact with authorities after flight arrival, recall bias caused by the length of time between flight arrival and survey response; and potential for contracting an ILI postarrival from a source other than the flight. Cross-checking of data collected by local health authorities at the time of flight arrival showed no recall bias. Response levels from passengers contacted by health authorities were higher than those not contacted, thereby limiting response bias. The spectrum of signs symptoms of passengers contracting ILI or pandemic (H1N1) 2009 varies; therefore, if a passenger did not return the survey or contact medical personnel or health authorities after the flight, some cases may have been missed (*28*). Media coverage of the arrival of flight 1 requesting passengers with ILI to contact health authorities was substantial. Although many passengers may be assumed to have then sought medical advice, the number of passengers who were not tested but reported ILI symptoms on their survey indicated this assumption was incorrect. Contracting pandemic (H1N1) 2009 postflight from a source in the community was unlikely. There was a small chance of contracting the disease preflight because of community transmission for flight 1; however, flight 2 originated from an area with no documented community transmission. At the time of investigation, community transmission of pandemic (H1N1) 2009 was not documented at the arrival port. The likelihood of community transmission is also low because all passengers with confirmed pandemic (H1N1) 2009 had symptom onset date within 48 hours after flight arrival.

Spread of pandemic (H1N1) 2009 and other ILIs occurred in limited zones of the aircraft during international flights into Australia during May 2009. The time required to contact passengers postflight resulted in the potential spread of disease into the community despite guidelines and policies in place to reduce the risk for disease importation. Nonetheless, application of these policies by Australian authorities may have assisted in delaying the importation of identified pandemic (H1N1) 2009 cases during the first month of the recent pandemic. The findings of this investigation suggest that efforts to prevent importation of respiratory diseases into a community and protection of individuals from in-flight exposure to ILI may require changes in international policies of both exit screening of symptomatic passengers preflight and contact tracing of those exposed to an ILI in flight. Further research on transmission of ILI in aircraft and into the effects of exit screening at international airport hubs to restrict travel of passengers with symptoms before flying would be of particular interest for respiratory disease of greater severity than pandemic (H1N1) 2009.
